# Editorial: Gut Microbial Response to Host Metabolic Phenotypes

**DOI:** 10.3389/fnut.2021.817501

**Published:** 2022-01-13

**Authors:** Jie Yin, Yong Su, Hui Han

**Affiliations:** ^1^College of Animal Science and Technology, Hunan Agricultural University, Changsha, China; ^2^College of Animal Science and Technology, Nanjing Agricultural University, Nanjing, China; ^3^State Key Laboratory of Animal Nutrition, Institute of Animal Science, Chinese Academy of Agricultural Sciences, Beijing, China

**Keywords:** gut microbiota, metabolism, disease, probiotics, diets

It is increasingly apparent that gut microbiota perform functions crucial to the host, such as regulating host physiology and influencing host health (1–3). Using fecal bacteria transplantation technology, Wu et al. found that fecal microbiota from obese Jinhua pigs and lean Landrace pigs exert different lipid metabolic phenotypes. Zheng et al. also witnessed gut microbial alterations in high fat diet-fed mice, with a high ratio of Firmicutes to Bacteroidetes and abundance of *Allobaculum*. Although this Research Topic failed to receive any papers about the role of gut microbiota in amino acid metabolism, nucleic acid metabolism, and carbohydrate metabolism, other reports have confirmed these functions (4, 5).

Currently, the gut microbiota is attracting much interest due to its role in maintaining host health and its association with all aspects of health and diseases. In this Research Topic, gut microbial disorders are screened in persistent atrial fibrillation patients (Xu et al.) renal cell carcinoma metastasis patients (Dai et al.), and a spinal cord injury animal model (Rong et al.). Xu et al. thoroughly discussed the taxonomic and functional characteristics of the gastrointestinal microbiota and demonstrated the profound relationship between gastrointestinal microbiota and metabolic disorders in ruminants. Together, these results further confirmed the role of gut microbiota in disease occurrence and development and manipulation of gut microbiota might, therefore, be considered a potential target for treating diseases.

Indeed, various disease treatment measures include gut microbial improvement, such as dietary probiotics (6). For example, Zhang et al. reported that dietary *Lactobacillus acidophilus* ATCC 4356 improved gut microbiota distribution and alleviated renal ischemia–reperfusion injury. Similarly, beneficial effects of *Lactobacillus* have been identified in lumbar disc herniation (Wang et al.), hypercholesterolemic golden hamsters (Yang et al.), asthma, and *Clostridium perfringens* infection (Wang et al.). In animal production, the gut is generally disturbed by weaning stress, dietary toxins, and pathogen infections, thus dietary probiotics have been widely introduced to maintain a healthy gut and guarantee higher production performance (7). *Lactococcus lactis*, in this Research Topic, has been identified to improve gut function in piglets (Yu et al.). However, probiotics are not limited to *Lactobacillus*, some species of *Bifidobacterium, Escherichia coli, Enterococcus*, and *Saccharomyces* have long been used as probiotics to alleviate various diseases by changing gut microbiota compositions.

Gut microbiota diversity and compositions are highly associated with dietary fluctuations. Thus, dietary manipulation has also been used to target gut microbiota to regulate host physiology and metabolism. In this Research Topic, Qian et al. found that dietary dried citrus peel (Chenpi) improved gut microbiota compositions in high fat diet-fed mice. Li et al. concluded that maternal fiber nutrition during pregnancy regulated the health of offspring, and the response of the maternal intestinal microbes played an important role in intervening in the phenotype of sows and neonatal piglets. Dietary protein and amino acids are the main factors shaping gut microbiota (8), Fu et al. also reported a role of tryptophan in gut microbiota. Besides, vitamin K2 (Liu et al.), β-carotene (Yuan et al.), olive fruit extracts (Wang et al.), bovine lactoferrin (Wang et al.), and β-sitosterol (Yu et al.) have been reported to shape gut microbiota compositions in this Research Topic.

How does gut microbiota affect host physiology and metabolism? Hou et al. showed that the gut–liver FXR–FGF19 axis is involved in *Lactobacillus delbrueckii*-promoted ileal bile acid deconjugation. In our previous studies, we found that gut microbiota-derived metabolites are highly associated with host metabolic reprograming (9–12). Furthermore, bacterial microRNA, bacteriocin, and microbiota sensing pathways have also been identified to be involved in the relationship between gut microbiota and host metabolism (13, 14). However, with the focus on the detailed mechanism by which gut microbiota influence host metabolism, much still needs to be elucidated.

In summary, papers from the current Research Topic screened the gut microbiota dysbiosis in various diseases and reported the beneficial roles of dietary probiotics and other active components in the improvement of gut microbiota. Despite the progress made in understanding the relationship between gut microbiota and host metabolism, there are a number of prominent research avenues that remain to be explored. For example, what are the molecular and physiological links between the gut microbiota and host metabolism at the epigenetic, transcriptome, and proteome levels? Gut microbiota is changed in various pathologic conditions and microbial biomarkers need to be screened in specific metabolic diseases. Additionally, dietary manipulation is widely used to maintain a healthy gut microbiota composition, and the interaction between diets, gut microbiota, and host metabolism will be an important area of future research.

## Author Contributions

All authors listed have made a substantial, direct, and intellectual contribution to the work and approved it for publication.

## Funding

This study was supported by the National Natural Science Foundation of China (32172761) and the Young Elite Scientists Sponsorship Program by CAST (2019-2021QNRC001).

## Conflict of Interest

The authors declare that the research was conducted in the absence of any commercial or financial relationships that could be construed as a potential conflict of interest.

## Publisher's Note

All claims expressed in this article are solely those of the authors and do not necessarily represent those of their affiliated organizations, or those of the publisher, the editors and the reviewers. Any product that may be evaluated in this article, or claim that may be made by its manufacturer, is not guaranteed or endorsed by the publisher.
